# Unveiling Unilateral Bell’s Palsy: A Case Series

**DOI:** 10.7759/cureus.64773

**Published:** 2024-07-17

**Authors:** Girish Suragimath, Disha G Suragimath, Ashwinirani SR

**Affiliations:** 1 Department of Periodontology, School of Dental Sciences, Krishna Vishwa Vidyapeeth (Deemed to be University), Karad, IND; 2 Department of Oral Medicine, School of Dental Sciences, Krishna Vishwa Vidyapeeth (Deemed to be University), Karad, IND; 3 Department of Radiology, School of Dental Sciences, Krishna Vishwa Vidyapeeth (Deemed to be University), Karad, IND

**Keywords:** artificial tears, antiviral treatment, systemic steroids, facial nerve palsy, facial palsy

## Abstract

Bell’s palsy is an idiopathic and uncommon peripheral nerve palsy that affects the facial nerve, leading to an inability to control the muscles of facial expression on the affected side. This paper presents two cases of unilateral Bell’s palsy in female patients treated with systemic steroids, antiviral drugs, and artificial tear substitutes. The treatment outcomes, clinical course, and recovery timelines are discussed in detail. A review of the current literature on the etiology, diagnosis, and management of Bell’s palsy is also provided to contextualize these cases within broader clinical practice.

## Introduction

Bell's palsy is an unexpected, idiopathic condition where the muscles on one side of the face become paralyzed or weak. This condition arises from a disorder of the seventh cranial nerve, also called the facial nerve, which is responsible for controlling facial muscle movements. The famous Scottish surgeon, Sir Charles Bell, named the condition Bell’s palsy [1]. Unilateral Bell’s palsy occurs in 70% of cases, with an incidence of approximately 25 per 100,000 people. In contrast, bilateral Bell’s palsy is very rare, with an incidence of 1 per 5,000,000 people [2]. Bell’s palsy affecting the pediatric group of patients has also been recorded in the literature [3].

The precise reason for Bell's palsy remains unknown, but it is believed to be the result of inflammation and swelling of the facial nerve. Potential triggers include viral infections such as herpes simplex virus (HSV), which is often implicated in nerve inflammation; it is also called Ramsay-Hunt syndrome [4]. The common predisposing factors are trauma to the maxillofacial region, cranial fractures, post-surgical palsy, Epstein-Barr virus infection, Lyme disease, otitis media, meningitis, syphilis, diabetes, neurological conditions like stroke, multiple sclerosis, Parkinson’s disease, vasculitis, and exposure to extreme cold [5-7]. The present article reports on two female patients with unilateral Bell’s palsy and their clinical features and treatment. A review of the current literature on the causes, diagnosis, and management of Bell’s palsy is also provided to familiarize these cases with broader clinical practice.

## Case presentation

Case 1

**Figure 1:**
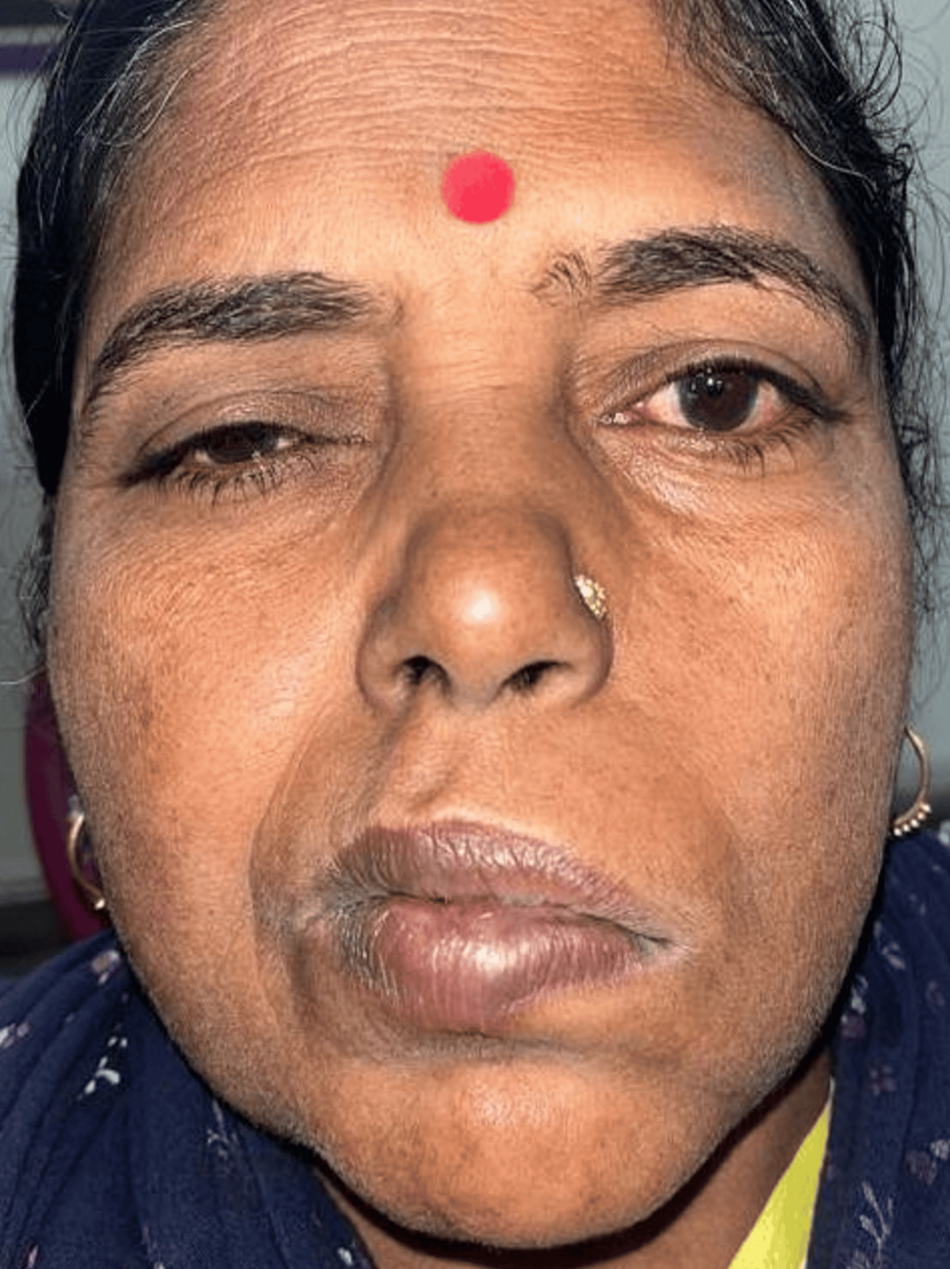
Deviation of the lower third of the face and tip of the nose to the right side

A 45-year-old female patient presented to the outpatient section, reporting typical facial asymmetry from the past month. The history revealed a change in facial symmetry and difficulty chewing for one month. Her medical history revealed that she was hypertensive and under medication for five years. The clinical examination of the patient revealed an obvious disfiguring facial asymmetry with facial expressions. The lower third of the face was deviated to the right side of the face (Figure 1).

**Figure 2:**
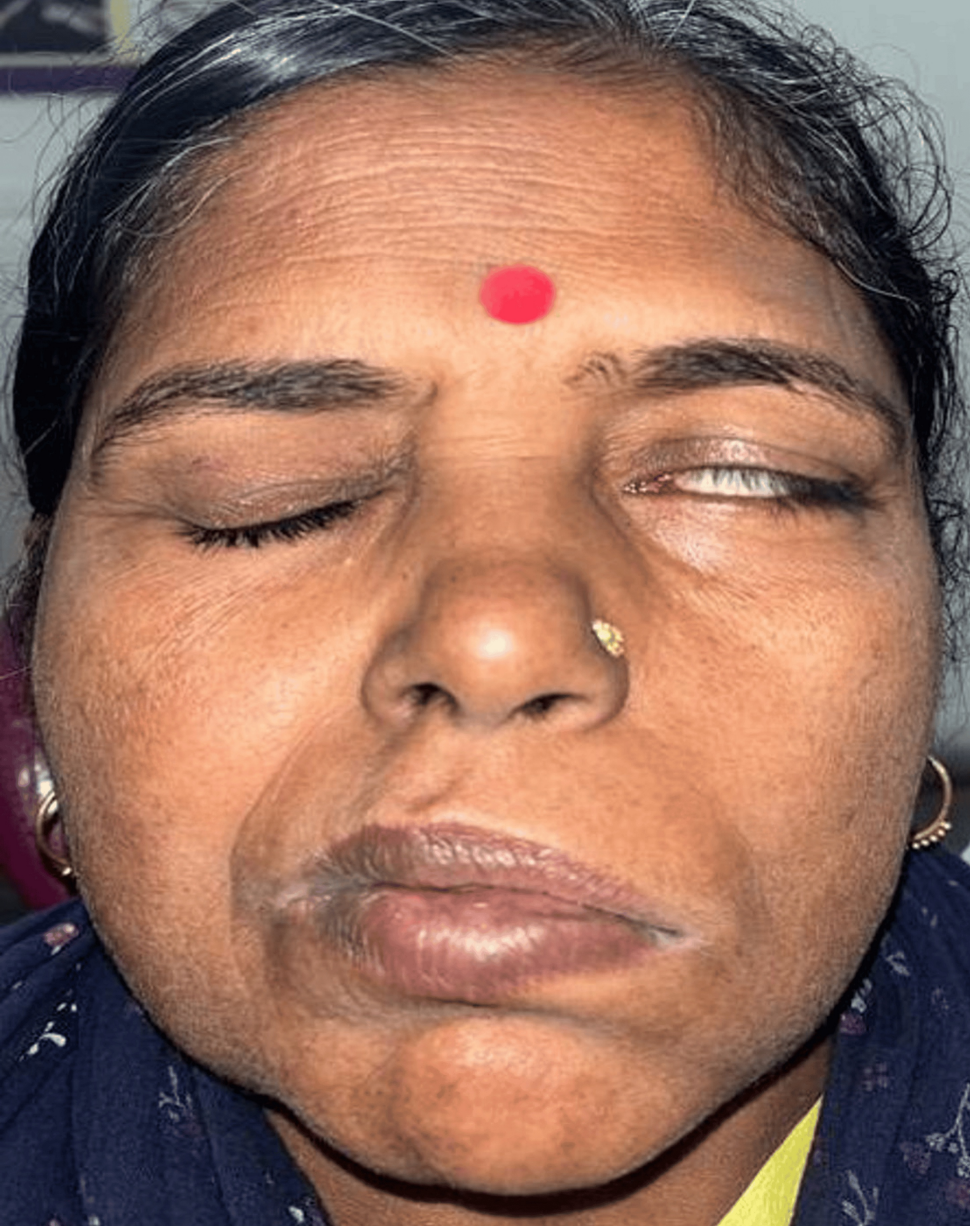
Incomplete closure of the left eye

The tip of her nose was pulled toward the unaffected right side. The cranial nerves were assessed, and reduced wrinkling on frowning was noticed on the left side of the forehead (Bell’s phenomenon), with incomplete closure of her left eye (Figure 2).

When she smiled and puffed her face, the muscle activity on the left side was noticeably decreased compared to the right side. Sensory examination revealed no sensory deficits on both sides of the face. Based on the clinical features, the provisional diagnosis of Bell’s palsy affecting the left side was attained.

The biochemical, hematological, and liver function laboratory investigations were within the normal limits. A CT of the brain was carried out, and no obvious abnormalities were detected. The final diagnosis of Bell’s palsy grade V, affecting the left side of the face as per the House-Brackmann facial nerve grading system, was arrived at.

The treatment plan included a systemic steroid (Tab Wysolone 20 mg) thrice a day with a tapering dose for 10 days, Tab Acyclovir 400 mg five times a day for 10 days, and artificial tears during the day for two weeks. The additional treatment of facial physiotherapy exercises combined with warm water compression was advised. To prevent conjunctival dryness, the patient was asked to keep the left eye shut with a sleep mask or tape at night. The patient was recalled after one month for a checkup, and it was observed that the patient had facial symmetry with complete closure of the left eye. The patient has been symptom-free since then, and no recurrence has been noted during her follow-up visits.

Case 2

**Figure 3:**
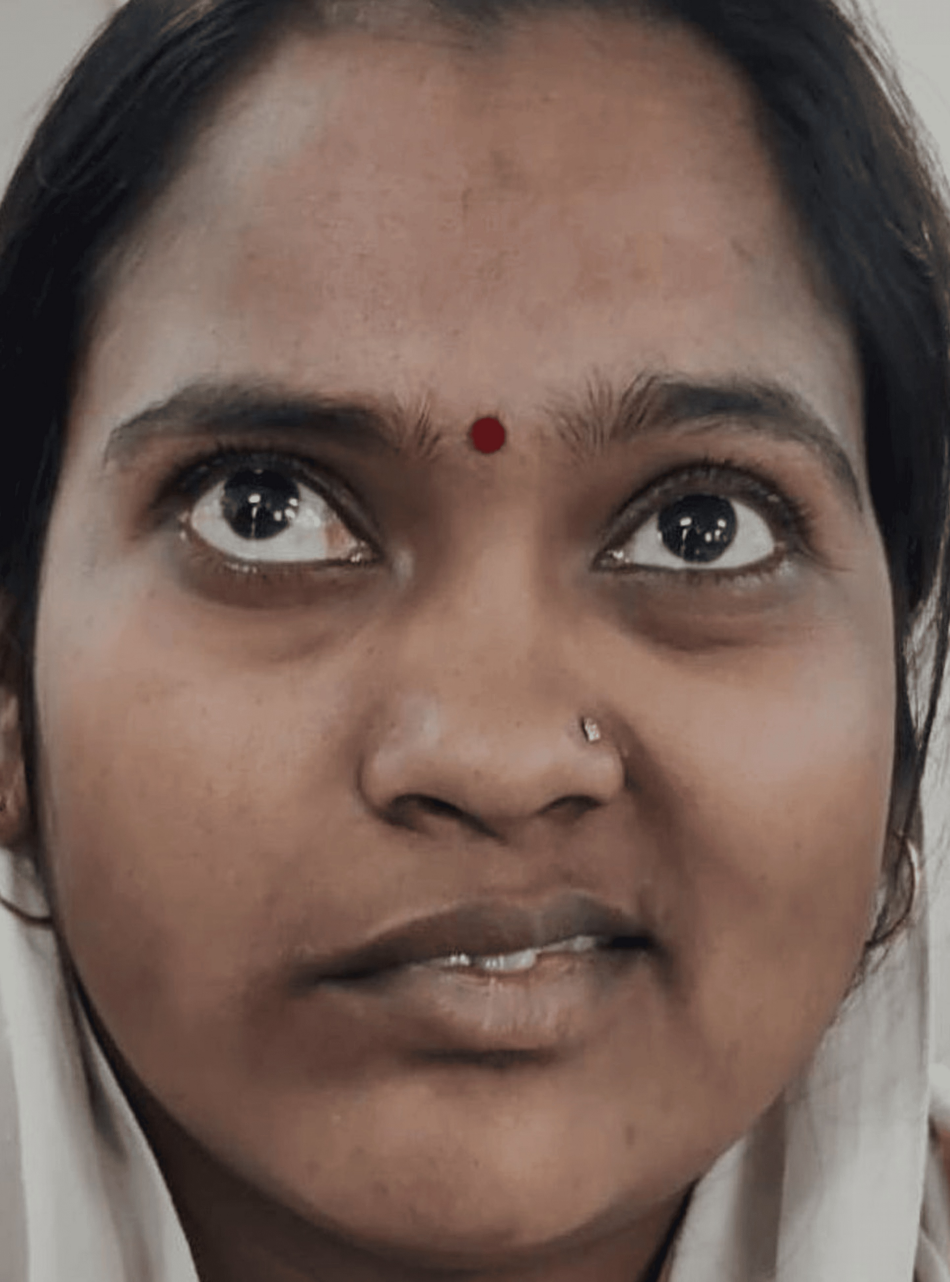
Deviation of the lower third of the face and to the left side

A female patient 30 years of age visited the outpatient department, reporting typical facial asymmetry, for 20 days. Her past medical history revealed the occurrence of recurrent vesicles on the right side of her lower lip. The clinical examination of the patient revealed moderate disfiguring of facial asymmetry with facial expressions. The corner of the mouth was deviated to the left side of the face. The tip of the nose was not deviated to the left side (Figure 3).

**Figure 4:**
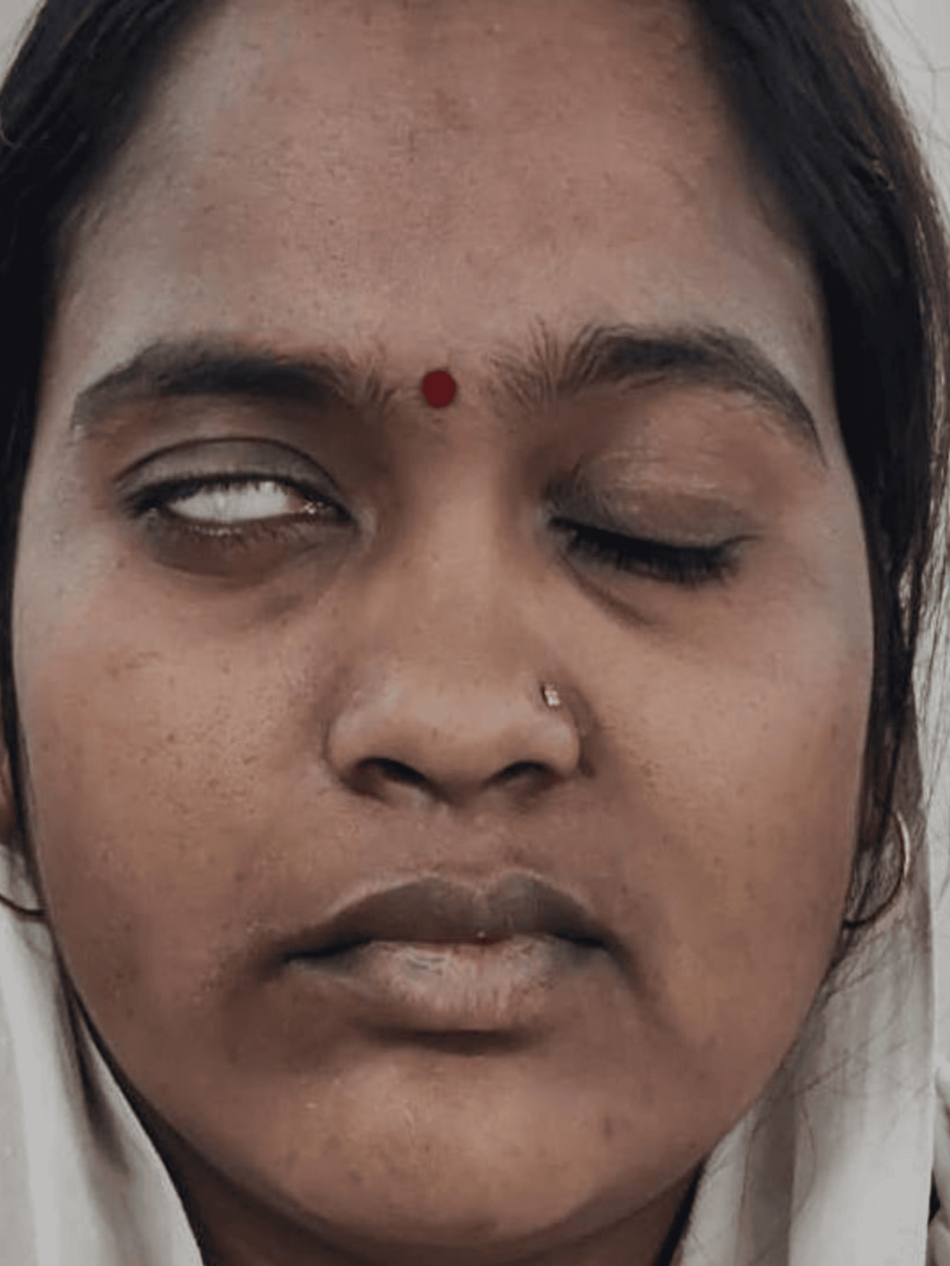
Incomplete closure of the right eye

The absence of wrinkling or frowning was noticed on the right side of the forehead. Bell’s phenomenon, with an incomplete closure of her right eye, was noticed (Figure 4).

While smiling and puffing her cheeks, she experienced reduced muscle activity on the right side of her face compared to her left side. Based on the above features, the diagnosis of Bell’s palsy grade IV, affecting the right side of the face according to House-Brackmann's facial nerve grading system was reached.

The treatment plan included a systemic steroid (Tab Wysolone 20 mg) thrice a day with a tapering dose for 10 days, Tab Acyclovir 400 mg five times a day for 10 days, and artificial tears during the day for two weeks. Facial physiotherapy exercises combined with warm water compression were initiated. To prevent conjunctival dryness, the patient was asked to keep the right eye shut with a sleep mask or tape at night. The patient was recalled after one month, and a good prognosis with facial asymmetry and closure of the right eye was observed.

## Discussion

The common etiology for unilateral facial weakness is stroke and Bell’s palsy; differentiating between them through early diagnosis and management will help the patient’s prognosis and recovery. Bell’s palsy, or idiopathic facial palsy, is characterized by sudden, temporary paralysis or weakness of the muscles, unilaterally on one side of the face. It occurs due to inflammation of the facial nerve at the geniculate ganglion, which leads to compression, ischemia, and demyelination of the nerve. Understanding Bell's palsy is crucial for early diagnosis and effective management, leading to better outcomes and faster recovery for patients. The salient features of Bell’s palsy are mentioned in Table 1.

**Table 1:** Salient features of Bell’s palsy [8-10]

Key point	Salient features
Incidence and prevalence	Unilateral Bell’s palsy: occurs in 70% of cases, with an incidence of approximately 25 per 100,000 people annually
	Bilateral Bell’s palsy: very rare, with an incidence of 1 per 5,000,000 people
Key characteristics and symptoms	Sudden onset: symptoms typically appear rapidly, often within hours to days
	Unilateral facial weakness: the hallmark of Bell's palsy is complete paralysis or weakness on the affected side of the face
	Inability to control facial muscles: affected individuals may struggle with blinking, smiling, or other facial movements on the impacted side
	Drooping of the mouth: one side of the mouth may droop, leading to difficulties with eating and speaking
	Loss of taste: some patients may experience taste loss on the anterior part tongue
	Hyperacusis: increased sensitivity to sound may occur in the affected ear
	Dryness of the eye and mouth: due to reduced control over the muscles that close the eye and secrete saliva
Causes and risk factors	Exact cause unknown: often linked to viral infections, such as the herpes simplex virus
	Triggers: stress, trauma, or upper respiratory tract infections can precede onset
Diagnosis	Clinical examination: observation of facial asymmetry and muscle weakness
	Imaging and tests: sometimes used to rule out other causes, such as stroke or tumors
Treatment	Medications: corticosteroids are commonly prescribed to reduce inflammation and swelling
	Physical therapy: exercises to strengthen facial muscles
	Protection of the eye: using eye drops or an eye patch to prevent drying if the eye cannot be closed properly
Prognosis	Recovery: most patients begin to improve within a few weeks and recover completely within three to six months
	Recurrence: rare, but possible in some cases
Pediatric Bell's palsy	Incidence: Bell’s palsy also affects the pediatric population, as documented in medical literature
	Symptoms and treatment: similar to those in adults, with emphasis on protecting the affected eye and ensuring proper nutrition and hydration

The House-Brackmann grading system, used to assess the degree of nerve damage, is classified into six types [11]: Grade I: normal facial function. Grade II: mild dysfunction with slight weakness and synkinesis. At rest, normal tone and symmetry are noted, and the motion of the forehead is good to moderate. Complete closure of the eye is achieved with minimal effort, but a slight asymmetry of the mouth is evident. Grade III: moderate dysfunction, which is obvious, yet no disfiguring alterations are observed between both sides of the face. Noticeable synkinesis, which is not severe, contracture, or hemifacial spasm, is observed. At rest, normal tone and symmetry are noted, and the motion of the forehead is slight to moderate. Complete eye closure can be achieved with effort, and slightly weak mouth movement is observed with maximal effort. Grade IV: moderately severe dysfunction, with obvious weakness and/or disfiguring asymmetry. At rest, normal tone and symmetry are noted, with no forehead motion. Incomplete eye closure and asymmetrical mouth opening are noted with maximal effort. Grade V: severe dysfunction, characterized by asymmetry of the face at rest. No forehead motion is observed, with incomplete eye closure. Grade VI: total paralysis with gross asymmetry.

In our report, both patients were female, of whom one had a history of recurrent herpes labial infection. As per one of the hypotheses, HSV remains dormant in the geniculate ganglion and gets reactivated by precipitating factors, starting to replicate and causing inflammation in the region. The inflammation in the geniculate ganglion and the labyrinthine segment of the facial nerve leads to entrapment and ischemia, ultimately resulting in neurapraxia or degeneration of the facial nerve distal to the mental foramen [12].

In the majority of cases, the cause is idiopathic, and patients improve completely without any therapy in three weeks [13]. In our study, both patients were treated with systemic steroids and antiviral drugs with artificial tears, and a good prognosis was noted. A differential diagnosis of otitis media, brain tumors, parotid neoplasm, cholesteatoma, and cerebral infarct could be considered for Bell’s palsy. Early diagnoses and proper management of Bell's palsy help the patient achieve normal function and better aesthetics.

## Conclusions

Bell's palsy is a sudden and often alarming condition that is usually idiopathic or related to a viral illness. Unilateral facial palsy can mimic a stroke, which is a life-threatening condition observed more frequently in women. Prompt treatment and follow-up in the majority of cases lead to full recovery. Ongoing research should aim to better understand the underlying causes and improve management strategies. Emergency physicians should be aware of the various diagnostic possibilities and the appropriate remedial measures to treat Bell’s palsy effectively.
